# Polymeric Giant Unilamellar Vesicles Support Longevity of Native Nuclei in Protocells

**DOI:** 10.1002/smsc.202400622

**Published:** 2025-03-18

**Authors:** Lukas Heuberger, Arianna Balestri, Shabnam Tarvirdipour, Larisa E. Kapinos, Roderick Y. H. Lim, Emanuel Lörtscher, Cora‐Ann Schoenenberger, Cornelia G. Palivan

**Affiliations:** ^1^ Department of Chemistry University of Basel Mattenstrasse 22 4058 Basel Switzerland; ^2^ Biozentrum and Swiss Nanoscience Institute University of Basel Spitalstrasse 41 4056 Basel Switzerland; ^3^ IBM Research Europe–Zürich Säumerstrasse 4 8803 Rüschlikon Switzerland; ^4^ NCCR ‐ Molecular Systems Engineering Mattenstrasse 22 4002 Basel Switzerland; ^5^ Swiss Nanoscience Institute (SNI) University of Basel Klingelbergstrasse 80 4056 Basel Switzerland

**Keywords:** native nuclei, nuclear delivery, peptide multicompartment micelles, polymeric GUVs, protocells

## Abstract

Protocells offer a versatile material for dissecting cellular processes and developing simplified biomimetic systems by combining biological components with synthetic ones. However, a gap exists between the integrity and complex functionality of native organelles such as nuclei, and bottom‐up strategies reducing cellular functions within a synthetic environment. Here, this gap is bridged by incorporating native nuclei into polymeric giant unilamellar vesicles (pGUVs) using double‐emulsion microfluidics. It is shown that the nuclei retain their morphology and nuclear envelope integrity, facilitating the import of co‐encapsulated peptide‐based multicompartment micelles (MCMs) via nuclear localization signals (NLS). Importantly, it is demonstrated that the nuclear import machinery remains functional inside the protocells, and by enriching the GUV interior with nuclear import‐promoting factors, the delivery efficiency of NLS‐MCMs significantly increases. The findings reveal that nucleated protocells preserve nuclear function and integrity for extended periods, providing a new platform for studying nuclear processes in a simplified, yet biologically relevant, environment. This approach opens avenues for creating advanced biohybrid materials, offering opportunities to investigate organelle behavior and their interactions with cellular components in greater detail. The findings establish a foundation for high‐throughput applications in synthetic biology and contribute valuable insights into sustaining complex cellular functions in engineered systems.

## Graphical abstract

Native nuclei are isolated and incorporated into polymeric giant unilamellar vesicles (pGUVs) using microfluidics. Nucleated pGUVs retain nuclear integrity and function, providing a new platform to study the import process of peptide‐based multicompartment micelles via nuclear localization signals. This bottom‐up chimeric system lays the groundwork for innovative bioapplications, such as high‐throughput screening of nuclear delivery.

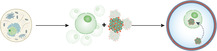

## 1 Introduction

Protocells emulate specific structures and functions of natural cells and play a vital role in bioinspired biotechnology.^[^ ^1^, ^2^, ^3^ ^]^ By abstraction and reduction of intricate, tightly regulated cellular processes, protocells help researchers to specifically address selected aspects of living cells. In this context, bottom‐up strategies, which start with individual building blocks to design synthetic cell‐like, offer the distinct advantage of providing controlled and simplified conditions at a molecular level. They facilitate both a deeper understanding of various cellular functions and processes, as well as the development of advanced systems for bioapplications.^[^ ^3^, ^4^ ^]^ Protocells have been mainly developed by combining synthetic compartments with biomolecules (including enzymes,^[^ ^5^ ^]^ proteins,^[^ ^6^ ^]^ and catalytic mimics^[^ ^7^ ^]^) either as a single functional component^[^ ^8^ ^]^ or acting in tandem inside.^[^ ^9^, ^10^ ^]^ A significant advancement has been made by encapsulating nano‐assemblies (such as liposomes, polymersomes, nanoparticles, and coacervates) within micrometer‐sized compartments that serve as cell‐mimicking templates, creating a compartment‐in‐compartment architecture that more closely resembles the structure of actual cells.^[^ ^11^, ^12^, ^13^ ^]^ Multicompartimentalization and supplementing biomolecules, either freely moving inside the cell‐like compartments^[^ ^14^ ^]^ or segregated in nanoassemblies that then act as artificial organelles,^[^ ^15^ ^]^ enhance the complexity of the protocells. Although including artificial organelles moves bottom‐up engineered systems a step closer to nature they do not match the complexity of cellular organelles. However, the integration of natural organelles into synthetic systems to study their behavior in a defined environment has only recently emerged.^[^ ^16^ ^]^ A small number of studies report the incorporation of whole native organelles, such as isolated mitochondria^[^ ^17^ ^]^ or chromatophores from *Rhodobacter sphaeroides* ^[^ ^18^ ^]^ into synthetic systems. Moreover, in case of native mitochondria, the hydrogel structure providing the synthetic cell‐mimetic environment is far from the cellular architecture mitochondria encounter in native cells.

Owing to their size and membrane structure, giant unilamellar vesicles (1–100 μm in diameter), assembled from amphiphilic molecules such as lipids, peptides, or amphiphilic block copolymers are well‐suited for integrating biological components to construct biohybrid protocells.^[^ ^19^, ^20^, ^21^ ^]^ Polymeric giant unilamellar vesicles, hereafter termed pGUVs, offer several advantages over their phospholipid counterparts, such as enhanced stability, limited permeability, tunable mechanical properties, and the ability to incorporate functional moieties that can mimic cellular features, including stimuli‐responsive behavior and chemical communication between compartments.^[^ ^3^, ^22^ ^]^ These features make pGUVs an ideal platform for studying organelles and their associated biological processes under various environmental and intra(proto)cellular conditions.^[^ ^4^ ^]^ In addition, the versatility of polymer chemistry allows for selecting giant unilamellar vesicles (GUV) membrane properties to control interactions at biointerfaces, particularly to minimize interactions with artificial and natural organelles, which is crucial for dynamic processes to take place. To the best of our knowledge, while one study has reported the encapsulation of native organelles within lipid GUVs,^[^ ^18^ ^]^ no studies have yet explored the encapsulation of nuclei inside GUVs or the incorporation of native organelles within polymeric GUVs (pGUVs).

Here, we fill this gap by introducing protocells consisting of a native single‐isolated nucleus residing in a pGUV generated via high‐throughput and ‐precision microfluidic technology. Microfluidics has proven to be a straight‐forward method for the controlled loading of GUVs with a wide range of cargoes, from soluble enzymes^[^ ^13^, ^23^ ^]^ to larger nanosized particles or even living bacteria.^[^ ^20^ ^]^ However, it has not yet been applied for the loading of natural organelles. We selected the nucleus as model organelle, as it serves as the repository of genetic information as the cell's control center. In eukaryotic cells, the nucleus is a specialized compartment, separated from other cellular components by the nuclear envelope, which consists of an inner and outer membrane. The distinct subcellular localization of many macromolecules, such as transcription factors, relies on the exchange of materials between the nucleus and the cytoplasm. This exchange is primarily facilitated by a transport system based on nuclear pore complexes (NPCs), which form channels in the nuclear envelope. The NPC facilitates two types of transport across the nuclear envelope: passive diffusion of small molecules and active, receptor‐mediated translocation of larger molecules containing a nuclear localization signal (NLS) or a nuclear export signal (NES).^[^ ^24^, ^25^ ^]^ This transport mechanism is recognized as a fundamental process in energy‐dependent cellular functions. Various neurodegenerative diseases are associated with a dysregulation of nucleocytoplasmic transport, highlighting its critical role in maintaining cellular homeostasis.^[^ ^26^, ^27^, ^28^, ^29^ ^]^ Consequently, understanding and manipulating nuclear transport is essential for advancing synthetic biology applications, particularly in developing therapeutic strategies to address dysfunctions.

Because isolated nuclei are prone to deterioration, we hypothesized that their encapsulation within cell‐sized pGUVs would increase their viability, allowing them to retain their function for extended time periods. In contrast to lipid‐based GUVs, the amphiphilic diblock copolymer poly(dimethylsiloxane)‐*block*‐poly(2‐methyl‐2‐oxazoline) (PDMS‐*b*‐PMOXA) has been demonstrated to form highly stable and impermeable vesicles of different sizes hosting various bottom‐up designed supramolecular assemblies in their aqueous cavity.^[^ ^19^, ^20^ ^]^ Using a double‐emulsion microfluidics approach, nucleated protocells were prepared from amphiphilic PDMS‐*b*‐PMOXA. The nuclei intended for encapsulation were isolated from a stable HeLa cell line constitutively expressing a histone–GFP fusion protein, which enables their tracking by fluorescence without the need for additional labeling. Accordingly, we examined key features, such as morphology and shape of the nuclei inside the pGUVs over time, as the integrity of nuclear architecture is essential for cell viability, and its alterations are hallmarks of various cellular processes, including senescence.^[^ ^26^, ^27^ ^]^

To investigate whether nuclei incorporated into protocells retain their ability to actively import macromolecules (larger than 40–60 kDa) through the NPC, we incorporated peptide nanocarriers exposing NLS, so called NLS‐multicompartment micelles (NLS‐MCMs), together with native nuclei into the pGUVs and examined their fate inside the nucleated protocells. These nanocarriers are based on an amphiphilic peptide termed NLS‐peptide, which is prone to self‐assembly and efficiently encapsulates genetic cargo through electrostatic interactions (antisense oligonucleotides, ASOs; DNA).^[^ ^30^, ^31^ ^]^ The NLS was included during the peptide synthesis as part of its hydrophilic domain such that the resulting nanocarriers expose NLS at their surface.^[^ ^30^ ^]^ These NLS‐MCMs have previously shown the ability to translocate to the nucleus through NPCs and deliver fluorescently labeled DNA to various human cancer cell lines.^[^ ^30^, ^31^ ^]^ NLS‐MCMs were incorporated into nucleated protocells as a “reporting” factor to evaluate the transport functionality of the nuclei. They are among the very few supramolecular nanoassemblies capable of nuclear transport and efficient payload delivery.^[^ ^32^ ^]^ The potency of the import machinery in nucleated protocells complemented with NLS‐MCMs was further explored by supplying factors that modulate the nuclear protein import cycle, thereby providing a more realistic cytosolic environment within the protocells. The resulting nucleated protocells have the unique advantage of shielding the encapsulated nuclei from potentially harmful external conditions while preserving their ability of nuclear transport within the protocell environment. This opens the possibility for using protocells in advanced investigations on interactions between the nucleus and cytoplasmic components. Beyond extending our knowledge of nuclear function within cells, this approach opens new pathways for bioapplications, such as high‐throughput screening of nuclear delivery.

## 2 Results and Discussion

### 2.1 Construction of Nucleated Protocells

**Figure 1:**
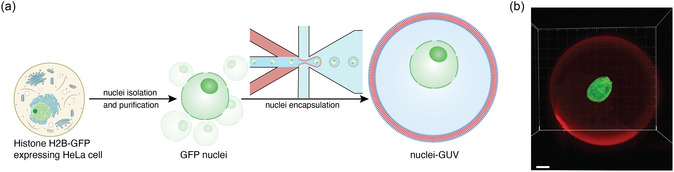
Workflow of nuclei encapsulation in pGUVs. a) Schematic representation of workflow used for creating chimeric nucleus‐pGUV cells. Nuclei isolated and purified from H2B‐GFP histone expressing HeLa cells are encapsulated in double emulsions by adding them to the inner aqueous phase prior to microfluidic droplet formation. Evaporation of the volatile organic phase yields pGUVs enclosing a nucleus. b) 3D‐reconstruction of a H2B‐GFP‐expressing nucleus (green) enclosed by an artificial cell membrane stained with BODIPY 630/650 (red). Scale bar, 5 μm.

For the construction of protocells hosting native nuclei, we made use of a double‐emulsion microfluidic system for the high‐throughput generation of double emulsions to obtain homogenous GUVs as protocell templates. A one‐step microfluidic droplet formation process based on a six‐way junction microfluidic chip was used to generate double emulsions composed of an inner aqueous buffer (IA), surrounded by a polymer‐containing middle phase (PO). An outer aqueous phase (OA) was used to break up fluids into monodisperse double‐emulsion droplets. The produced double emulsions were exposed to air to evaporate the volatile organic solvent layer, resulting in GUVs with a diameter of 38.5 ± 0.9 μm (±2.4%, Figure S1, Supporting Information) bounded by a single polymer bilayer. To take advantage of the high encapsulation efficiency offered by microfluidics, we isolated nuclei from H2B‐GFP histone expressing HeLa cells and added them to the IA (**Figure** **1**a).

Different dilutions of the nuclei in IA were tested to find a balance between the number of nucleated pGUVs being produced and the clogging of the microfluidic devices by aggregated nuclei. Considering a pGUV volume of 2.4 × 10^4^ μm^3^ and an initial cell number of 5 × 10^5^ cell mL^−1^ that was diluted 5‐fold, a maximum encapsulation rate of 0.03 nuclei pGUV^−1^ or 1 nucleus per 34 GUVs could be achieved theoretically. Experimentally, an encapsulation of 1 nucleus per 30 pGUV was determined, equivalent to an encapsulation efficiency of ≈89%. At an estimated nuclei concentration in the IA of 10^5^ nuclei mL^−1^, nuclei were successfully encapsulated in pGUVs while maintaining the rounded morphology typical for HeLa cell nuclei (Figure 1b).

For a detailed analysis of the nuclear morphology, we used three‐dimensional (3D) reconstructions of z‐stacked fluorescence images recorded by Airyscan confocal laser scanning microscopy (CLSM) followed by shape recognition (Figure S2, Supporting Information). Nuclei imaged in their native environment, (i.e., within living cells) served as a gold standard. The preparation of these protocells first required the isolation of the nuclei from living cells and then their incorporation into pGUVs by microfluidics. The procedure after obtaining the isolated nuclei until producing nucleated protocells via microfluidics took ≈4 h. Thus, we defined the endpoint of protocell production as our time zero (*t*_0_). Accordingly, the appearance, volume, and fluorescence of nuclei inside pGUVs were compared to those of isolated nuclei treated correspondingly, both at *t*_0_ at room temperature, and after 20 h incubation (*t*_20_) at physiological temperature (37 °C). Because nuclei inside cells (*in cellulo*) are subject to changes in shape and size, for example, due to cell cycle events, we sampled nuclei from >20 cells to obtain an average size and shape of a nucleus *in cellulo* that can be compared to the isolated and encapsulated nuclei.^[^ ^33^ ^]^

**Figure 2:**
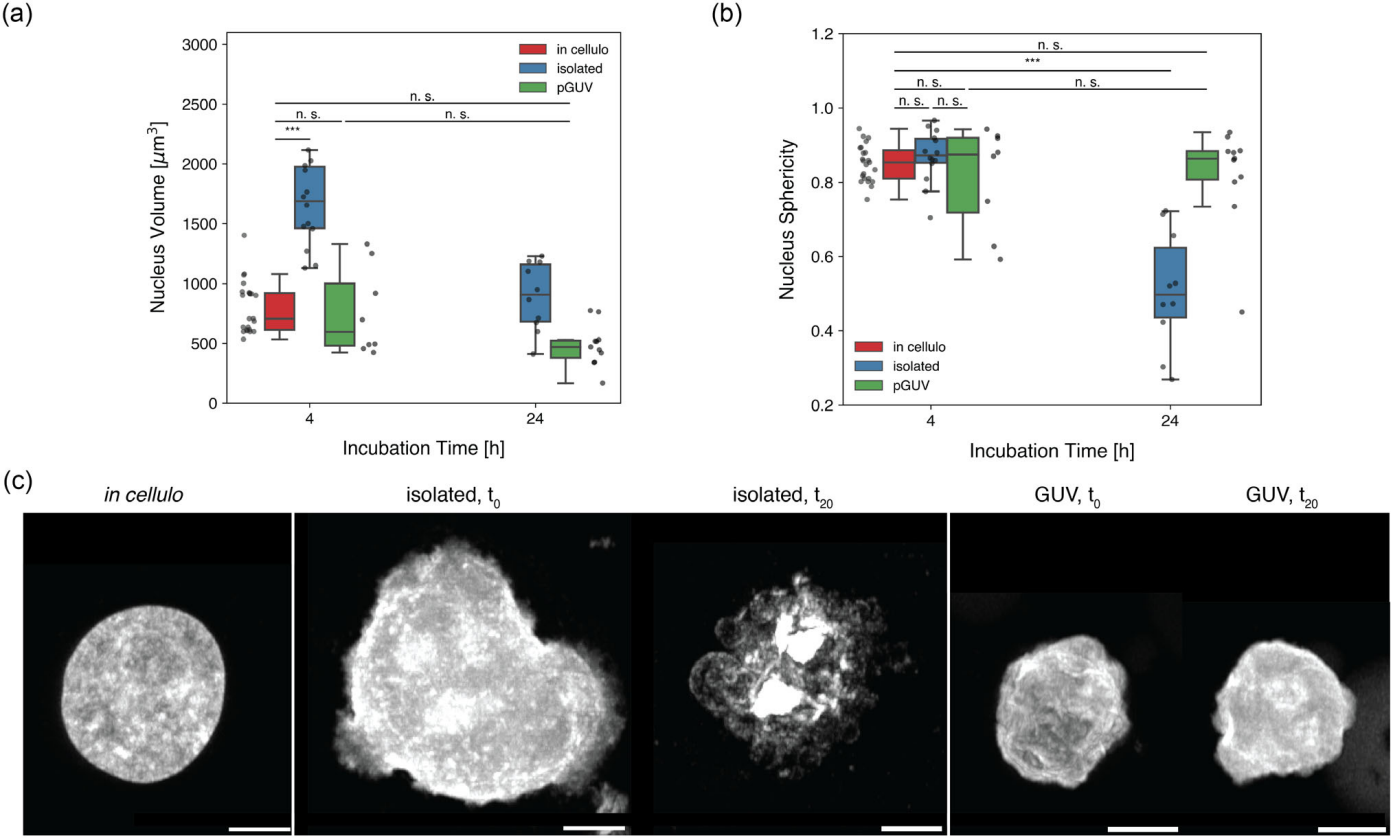
Time‐resolved morphology of isolated and pGUV‐enclosed nuclei. a) Comparison of nucleus volume *in cellulo*, isolated nuclei and nuclei encapsulated in pGUVs at *t*_0_ or *t*_20_ (*n* ≥ 8 nuclei/condition). b) Sphericity comparison of nuclei 4 h (*t*_0_) or 20 h (*t*_20_) post isolation (*n* ≥ 8 nuclei/condition). c) Representative micrographs showing maximal projections along the *z*‐axis of GFP‐fluorescent nuclei (b/w). Scale bars, 5 μm.

Compared to the average volume of nuclei *in cellulo* (804 ± 217 μm^3^), the isolation procedure resulted in a mean nuclear volume of 1795 ± 693 μm^3^ measured at *t*_0_, corresponding to a 123% volume increase. In contrast, when nuclei that were encapsulated inside GUVs were measured at the same time point, their volume (675 ± 437 μm^3^) showed no significant difference to that of nuclei in their native environment (**Figure** **2**a). Notably, the subsequent incubation of isolated nuclei at 37 °C resulted in disintegration of the nuclei together with a significant decrease in size over 20 h (Figure 2a,d), while the size of encapsulated nuclei remained stable (Table S1, Supporting Information).

A mean nucleus sphericity of 0.85 was measured *in cellulo,* which is similar to that observed for isolated and encapsulated nuclei (0.84 and 0.87 respectively, Figure 2b). Incubation of isolated nuclei at 37 °C caused a significant change in morphology toward a less round, more frayed shape, and a reduction in mean sphericity to 0.56, indicative of the deterioration of isolated nuclei over time. In contrast, nuclei residing inside GUVs showed no significant shape change over 20 h (sphericity 0.79, *p* = 0.98, Table S1, Supporting Information).

The nuclear laminas underneath the nuclear envelope are key regulators of nuclear morphology, structure, and mechanics.^[^ ^34^ ^]^ Because pGUV‐encapsulated nuclei showed no significant morphological changes over time, we conclude that the nuclear lamina remained largely intact both in the process of protocell preparation and over the following 20 h incubation at 37 °C.

The deterioration of isolated nuclei was also manifest as a 90% decrease in GFP fluorescence compared to nuclei *in cellulo*. However, due to the protective environment provided by the pGUVs, no significant change in fluorescence (−8.4%, *p* = 0.99) was observed in the encapsulated nuclei (Figure 2c,d and Table S1, Supporting Information). This data demonstrates that using microfluidics, pGUVs were readily equipped with a nucleus and provided a distinct, protective environment, potentially increasing the lifespan of native nuclei ex vivo.

### 2.2 Nuclear Translocation in Nucleated Protocells

**Figure 3:**
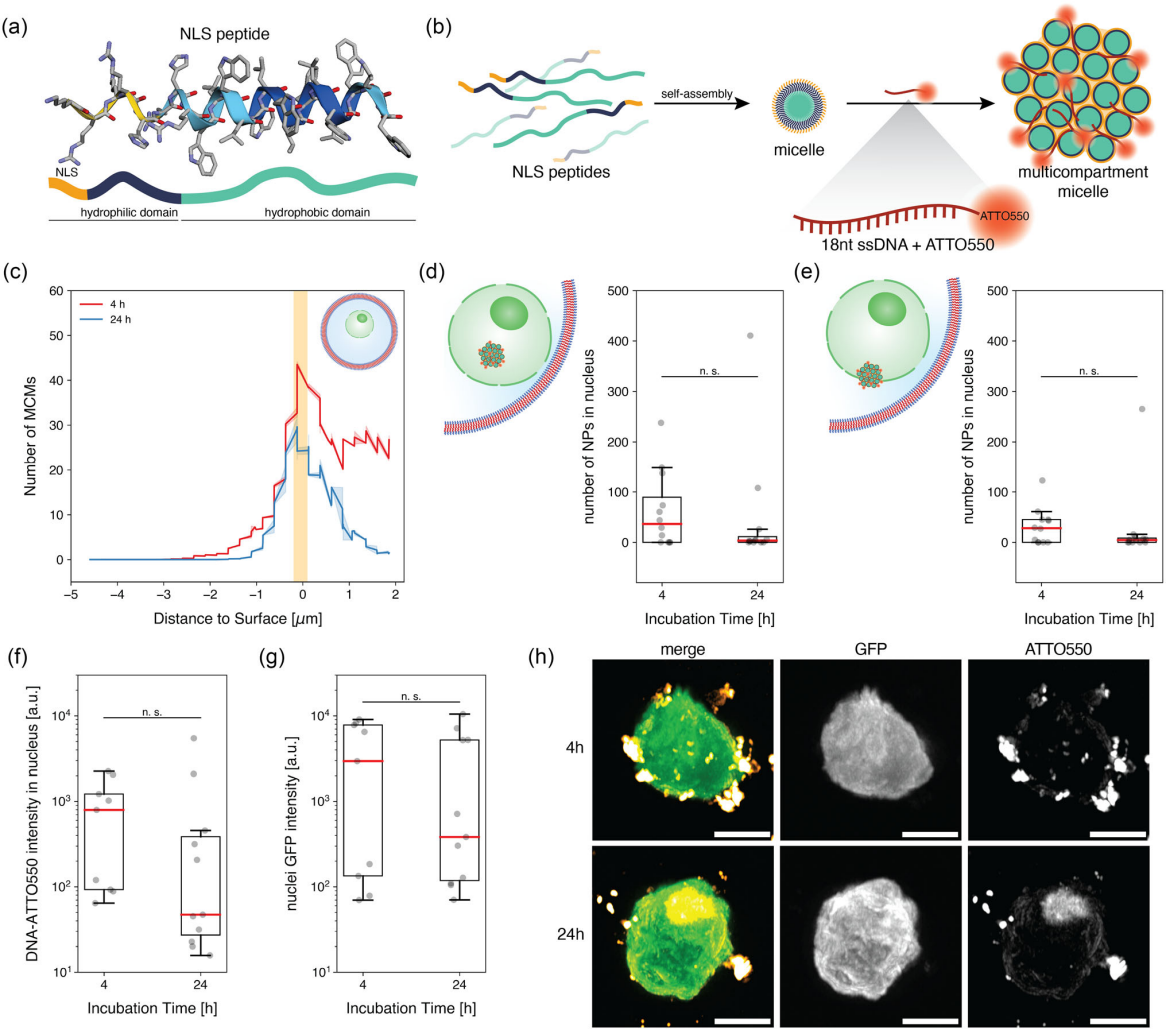
Nuclear trafficking of NLS‐DNA‐MCMs in nucleated protocells. a) Predicted 3D‐structure of amphiphilic NLS‐peptide. Structure prediction made with AlphaFold.^[^ ^47^ ^]^ b) Schematic representation of single and multicompartment micelle from self‐assembly of NLS‐peptides by solvent exchange. Fluorescently (ATTO550) labeled ssDNA is entrapped between the individual micelles of the multicompartment assembly through electrostatic interactions, resulting in fluorescent, nucleus‐targeting nanocarriers. c) NLS‐DNA‐MCM location in relation to the surface of the MCM‐nucGUVs at *t*_0_ or *t*_20_. The nuclear envelope region is indicated in orange. d) Number of NLS‐DNA‐MCMs inside the nucleus of MCM‐nucGUVs at *t*_0_ and *t*_20_. e) Number of NLS‐DNA‐MCMs associated with the nuclear envelope in MCM‐nucGUVs at *t*_0_ and *t*_20_. f) DNA‐associated ATTO550 fluorescence of MCM‐pGUVs at *t*_0_ and *t*_20_. g) GFP fluorescence of MCM‐nucGUV nuclei at *t*_0_ and *t*_20_. *n* ≥ 7 nuclei/condition. One‐way ANOVA was used for comparison: *p* > 0.05 (n.s.), *p* < 0.05 (*), *p* < 0.005 (**), *p* < 0.0005 (***), and n.s. = not significant, Tukey's post hoc test. h) Representative micrographs depicting maximal projections along the *z*‐axis of GFP‐fluorescent nuclei (green) and ATTO550‐DNA loaded‐NLS‐MCMs (yellow) in MCM‐nucGUVs over time. Single channels are depicted in black and white. Scale bars, 5 μm.

Creating nucleated protocells, henceforth termed nucGUVs, involves not only assessing the morphological appearance of the encapsulated nuclei but also their sustained functionality within the synthetic compartment. A major prerequisite for nuclear function is the nucleocytoplasmic transport mediated by NPCs.^[^ ^35^ ^]^ Hence, to assess nuclear functionality in protocells, the import of nucleus‐targeting nanocarriers was investigated. The nanocarriers, based on the self‐assembly of an amphiphilic peptide, are designed to target the nucleus through surface‐exposed NLS, which consists of a short sequence of amino acids (KRKR) (**Figure** **3**a and S3a, Supporting Information). NLS‐peptide self‐assembly in the presence of an 18 nucleotide single‐stranded DNA (ssDNA) coupled to a fluorescent dye (ATTO550, Note S2, Supporting Information) resulted in fluorescent, DNA‐loaded nanocarriers. Due to their spherical morphology and multi‐compartment micellar (MCM) architecture, as revealed by cryogenic electron microscopy (S4a, Supporting Information),^[^ ^30^, ^31^ ^]^ DNA‐loaded nanocarriers were named NLS‐DNA‐MCMs (Figure 3b). Dynamic light scattering measurements showed that NLS‐DNA‐MCMs had a hydrodynamic diameter (D_H_) of 52 ± 12 nm and a polydispersity index (PDI) of 0.19 (Figure S4b, Supporting Information). Zeta potential measurements indicated a surface charge of 25 ± 6 mV when the negatively charged DNA was entrapped. Based on brightness analysis of fluorescence correlation spectroscopy measurements, we calculated 18 ± 4 DNA molecules per nanocarrier.^[^ ^36^ ^]^ In addition, the fluorescent oligonucleotide cargo enabled tracking the journey of NLS‐DNA‐MCMs added to isolated nuclei in bulk as well as within the nucGUVs. Based on their fluorescence, the localization of nanocarriers was determined relative to the nuclear envelope and their penetration depth was quantified (Figure S5, Supporting Information).

We first investigated the nuclear translocation of NLS‐DNA‐MCMs in isolated nuclei to determine their import proficiency in bulk. Based on the 3D‐reconstruction of fluorescence recordings from individual nuclei, we localized the position of the fluorescent NLS‐DNA‐MCMs in relation to the nuclear envelope. To quantitatively assess nuclear import, we defined the nuclear envelope region as extending 0.2 μm inside and 0.1 μm outside of the detected nuclear membrane, and we determined the number of particles relative to this envelope location at *t*_0_ (NLS‐DNA‐MCMs freshly added to isolated nuclei) and *t*_20_ of co‐incubation at 37 °C (Figure S5a, Supporting Information). The total number of NLS‐DNA‐MCMs located inside the nuclei increased significantly over this time period, while the number of particles associated with the nuclear envelope region remained unchanged (Note S3 and Figure S5, Supporting Information). However, although the number of NLS‐DNA‐MCMs inside the nucleus increased over 20 h, the intrinsic GFP fluorescence of the corresponding nuclear volume decreased significantly (Figure S5f, Supporting Information). This data suggested that the accumulation of NLS‐DNA‐MCMs in the nucleus results from the deterioration of the latter. Conceivably, due to a potential disassembly of the nuclear envelope, NLS‐DNA‐MCMs may more readily enter the nucleus, where they can bind to residual proteins involved in transport, for example, importins.^[^ ^37^ ^]^

To address the import proficiency of nuclei incorporated in protocells, where nuclear morphology is preserved over 20 h, we used microfluidics to produce nucleated pGUVs loaded with NLS‐DNA‐MCMs (MCM‐nucGUVs). This was achieved by supplying the inner aqueous phase with both, NLS‐DNA‐MCMs and nuclei. 3D‐reconstructions of z‐stacked fluorescence images of the resulting protocells containing a GFP‐expressing nucleus and ATTO550‐labeled DNA‐loaded NLS‐MCMs were used to quantify their presence in the nucleus through colocalization (Figure 3c). Similar to the trend observed with isolated nuclei (Figure S5a, Supporting Information), MCM‐nucGUVs showed a colocalization of NLS‐DNA‐MCMs with the nucleus after 20 h of incubation (Figure 3c). However, compared to *t*_0_ of isolated nuclei, no significant differences were detected in the total number of NLS‐DNA‐MCMs associated with the nucleus (*p* = 0.30, Figure 3d,e). In a confined environment, the number of NLS‐DNA‐MCMs available for nuclear import is limited to the encapsulated nanocarriers, while in a bulk situation, the number of available NLS‐DNA‐MCMs is higher. The unrestricted availability of nanocarriers may have contributed to their increased nuclear accumulation in isolated nuclei.

Notably, the employed MCMs undergo temperature‐dependent dissociation over time^[^ ^30^, ^36^ ^]^ resulting in smaller MCMs and individual micelles that can readily import into the nucleus. Thus, quantifying both the number of NLS‐DNA‐MCMs and the fluorescent intensity of ATTO550‐DNA within the nucleus, are crucial to determine nuclear import efficiency. While the fluorescent intensity of ATTO550‐DNA in bulk increased significantly after 20 h of incubation compared to *t*_0_ (Figure S5, Supporting Information), in a confined scenario, no significant increase in ATTO550‐DNA fluorescence intensity was detected over the same incubation period (*p* = 0.68, Figure 3d). Furthermore, unlike non‐encapsulated nuclei (Figure S5, Supporting Information), neither the presence of NLS‐DNA‐MCMs nor the incubation time affected the intrinsic GFP fluorescence of the nuclei (*p* = 0.76, Figure 3f,h). In addition, encapsulation of NLS‐DNA‐MCMs and their nuclear trafficking appeared to have no influence on nuclear morphology (Figure S6, Supporting Information). This indicates that majority of NLS‐DNA‐MCM translocation into the nucleus takes place during microfluidic formation of corresponding protocells and is largely achieved at *t*_0_.

### 2.3 Transport Through the Nuclear Pore Complex of Nucleated Protocells

**Figure 4:**
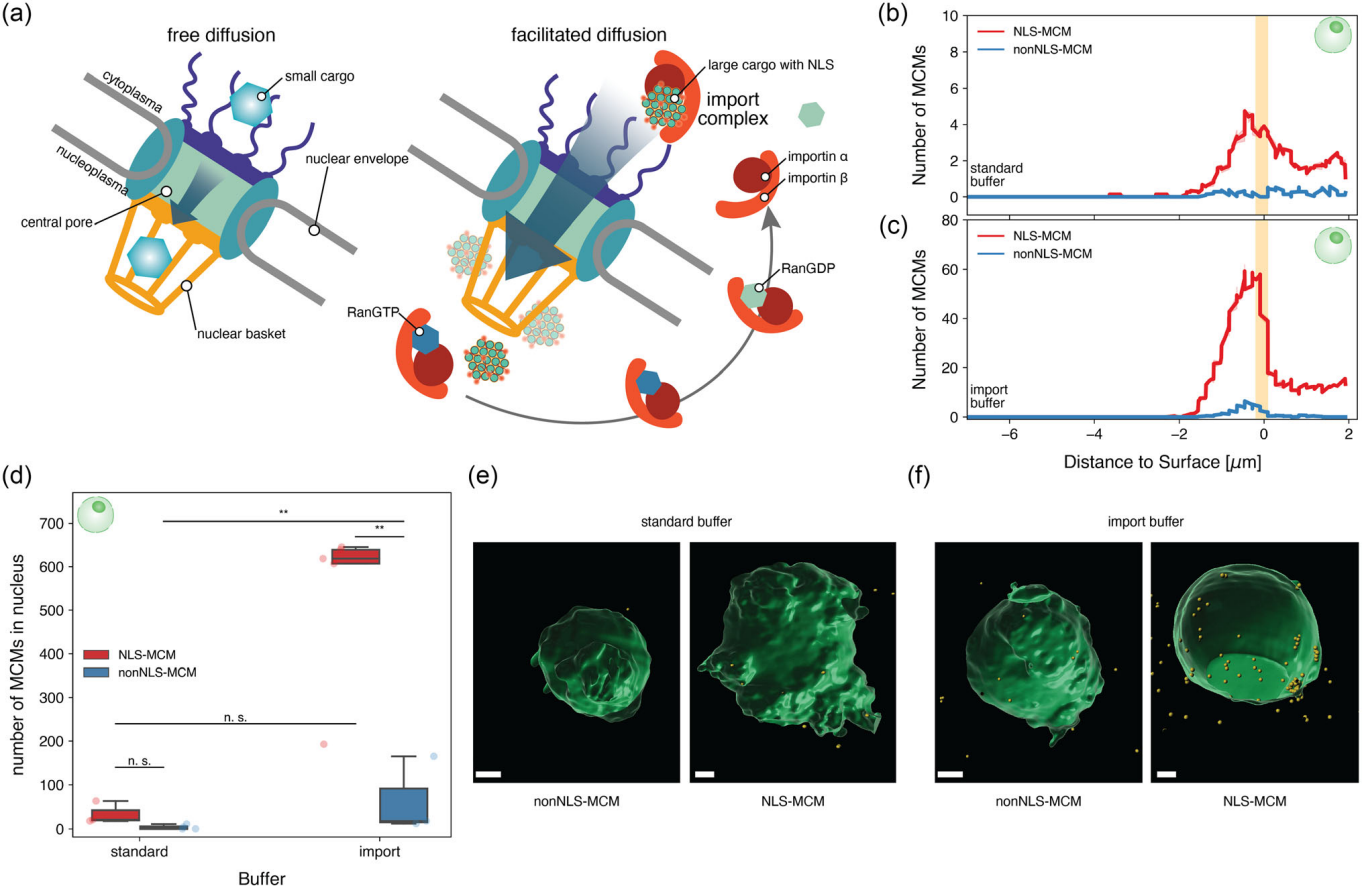
MCM Import through the nuclear pore complex in isolated nuclei. a) Schematic overview of two types of nuclear import; passive diffusion and active, chaperone‐mediated transport. Free diffusion allows ions and small proteins to enter the nucleus, whereas facilitated diffusion requires additional chaperones that mediate the interaction between the molecular cargo and the NPC to promote the import of larger structures. b,c) Localization of NLS‐ and nonNLS‐DNA‐MCMs with respect to the nuclear surface at *t*_0_ of incubation in standard buffer (b) and nuclear import buffer (c). The nuclear envelope region is indicated in orange. d) Total number of MCMs in isolated nuclei in standard and import buffer. *n* ≥ 3 nuclei/condition. Two‐way ANOVA was used for comparison: *p* > 0.05 (n.s.), *p* < 0.05 (*), *p* < 0.005 (**), *p* < 0.0005 (***), and n.s. = not significant, Tukey's post hoc test. e,f) 3D reconstructions of isolated nuclei (green) incubated with MCMs (yellow) with and without the NLS sequence in (e) standard buffer and (f) import buffer. Scale bars, 2 μm.

The NPC, embedded into the nuclear envelope, is one of the largest macromolecular assemblies in cells (**Figure** **4**a). Its central channel, with an inner diameter of ≈42.5 nm in humans,^[^ ^38^ ^]^ is the sole gateway for the bidirectional transport of small molecules to macromolecular complexes into and out of the nucleus.^[^ ^39^ ^]^ In most cases, the transport of large molecules into the nucleus is promoted by NLS and its interactions with import factors.^[^ ^40^ ^]^ Including an NLS in the amino acid sequence of the amphiphilic peptide was shown to significantly enhance the nuclear localization of NLS‐DNA‐MCMs, and their genetic cargo in living cells.^[^ ^30^ ^]^ NLS‐mediated trafficking into the nucleus is dependent on import factors such as importin‐α/β, adjuvant proteins and energy substrates (Figure 4a). One main energy source for nuclear transport is provided by the Ras family GTPase Ran, which cycles between a guanosine triphosphate (GTP), and a guanosine diphosphate (GDP) bound state.^[^ ^41^ ^]^ While isolated nuclei are likely to contain residual carrier proteins and adjuvants, supplementing them with additional energy donors and transport proteins increases the efficiency of facilitated transport across the nuclear membrane.^[^ ^42^ ^]^ To provide import‐promoting components in nucleated protocells, we used an “import buffer” as the inner aqueous phase in microfluidics. This buffer consisted of the standard inner aqueous phase (“standard buffer”) supplemented with GTP, ATP, RanGDP, and Nuclear Transport Factor 2 (NTF2), which facilitates the import of Ran, along with DTT as a reducing agent (for more detail see Section 4. Experimental, Fluid Phase Composition for Microfluidics).^[^ ^42^, ^43^ ^]^ Standard and import buffers were compared to explore the significance of the NLS in the nuclear import of MCMs into isolated nuclei and nucleated protocells.

Isolated nuclei were incubated with ATTO550‐DNA loaded MCMs assembled from NLS‐peptides (Figure S3a, Supporting Information) and peptides lacking an NLS, nonNLS‐peptides (Figure S3b and Note S1, Supporting Information) in standard buffer (Figure 4b) and in import buffer (Figure 4c) under the experimental conditions described earlier, that is, the time zero *t*_0_ corresponding to 4 h after adding them to isolated nuclei at room temperature (Figure 4b). For both buffers, an accumulation of MCMs below the nuclear envelope was observed in isolated nuclei for MCMs that exposed NLS on their surface compared to those lacking an NLS (nonNLS) (Figure 4b,c), indicating that the NLS was critical for an efficient translocation of the nanosized MCMs. Notably, the number of NLS‐DNA‐MCMs located at the nuclear face of the nuclear envelope was almost 10‐fold higher if nuclei and MCMs were incubated in import buffer instead of standard buffer (Figure 4c). This data indicates that the import buffer promotes the interactions of the NLS‐DNA‐MCMs with the import machinery in isolated nuclei. Incubation in import buffer also led to a small increase of nonNLS‐DNA below the nuclear envelope compared to standard buffer. However, when analyzing the number of MCMs throughout the entire nucleus rather than in the proximity of the nuclear envelope (Figure 4d), the increase of nonNLS‐DNA‐MCMs in import buffer was found to be not significant. As suggested by the accumulation of NLS‐DNA‐MCMs below the nuclear envelope, calculating the number of NLS‐DNA‐MCMs throughout the nucleus (≈50 and 600 NLS‐DNA‐MCMs with standard and import buffer, respectively) confirmed that the NLS significantly improved their import into the nucleus provided buffer components that promote the interaction of the NLS with the nuclear transport machinery are available (Figure 4d–f). An optical section through the mid‐plane of the nucleus directly resolved the inside versus outside distribution of MCMs (Figure S7, Supporting Information). Accordingly, in standard buffer, the difference between NLS‐ and nonNLS‐DNA‐MCMs was not significant. However, in isolated nuclei the buffer conditions had no significant effect on the import of nonNLS‐DNA‐MCMs, only on NLS‐DNA‐MCMs (Figure 4d).

**Figure 5:**
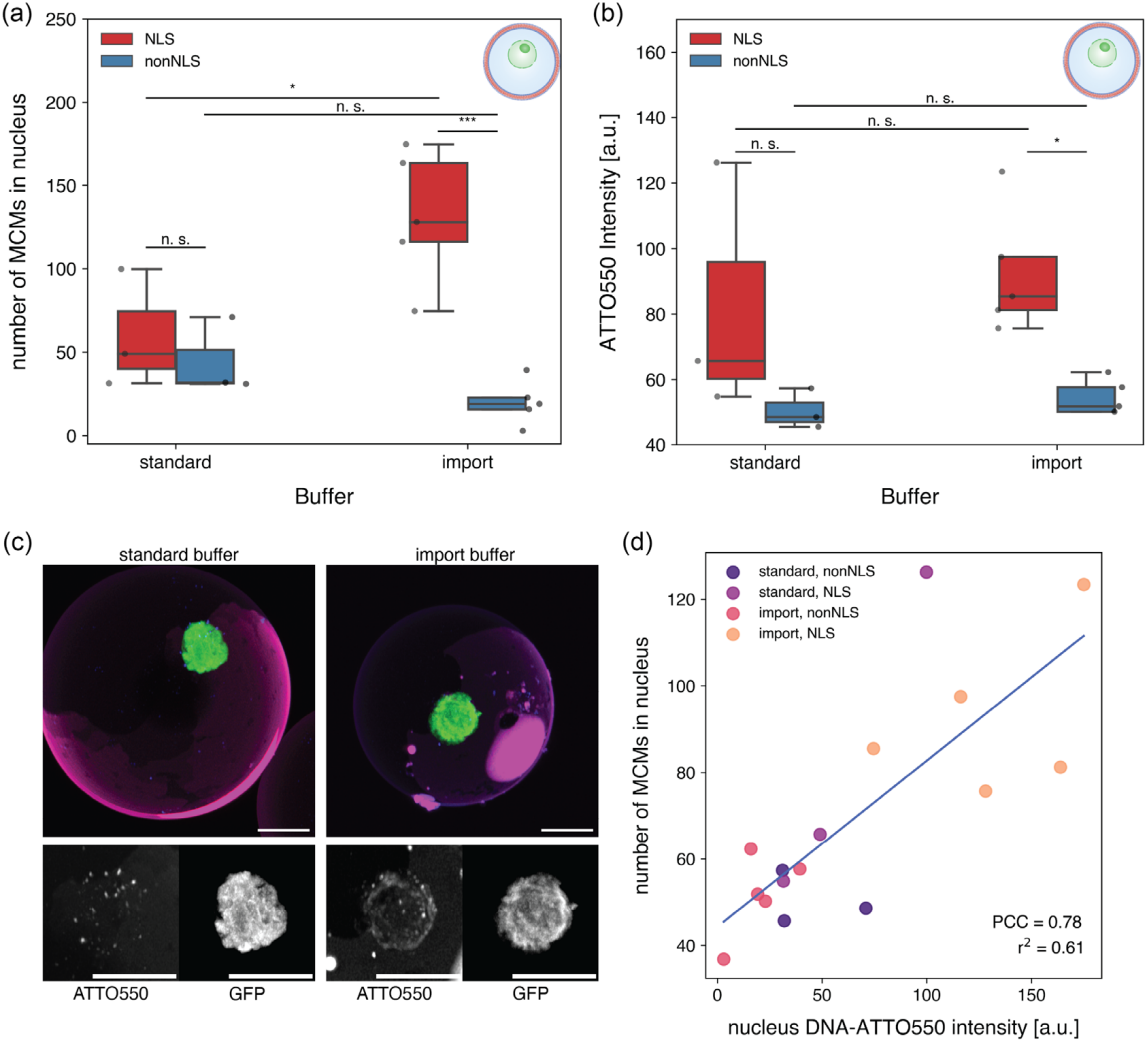
NLS‐mediated nuclear translocation of nanocarriers in MCM‐nucGUVs. a) Total number of MCMs in the nucleus of MCM‐nucGUVs in standard or import buffer. b) MCM‐based ATTO550 fluorescence of nuclei in MCM‐nucGUVs in standard or import buffer. *n* ≥ 3 nuclei/condition. Two‐way ANOVA was used for comparison: *p* > 0.05 (n.s.), *p* < 0.05 (*), *p* < 0.005 (**), *p* < 0.0005 (***), and n.s. = not significant, Tukey's post hoc test. c) Micrographs depicting maximal projections along the *z*‐axis of GFP fluorescent nuclei (green) and ATTO550‐DNA‐loaded MCMs (blue) in MCM‐nucGUVs (red) and zoom‐in of GFP and ATTO550 channels (black/white). Scale bars, 10 μm. d) Correlation between number of MCMs in the nucleus and ATTO550 fluorescence of the nucleus. Linear regression (blue line), *r* ^2^ = 0.61, Pearson correlation coefficient = 0.78.

After demonstrating that the import buffer improved the efficiency of NLS‐DNA‐MCMs nuclear translocation in isolated nuclei, we co‐encapsulated both nuclei and NLS‐DNA‐MCMs or nonNLS‐DNA‐MCMs in protocells using microfluidics, with either the standard or import buffer as the aqueous phase. We first verified by confocal microscopy that the different “cytosols”—standard or import buffer—inside the resulting NLS‐MCM‐nucGUVs and MCM‐nucGUVs, had no adverse effect on the stability of the loaded MCMs under the same experimental conditions used previously (Figure S8, Supporting Information). We then analyzed the import of the respective MCMs into the nuclei of the MCM‐nucGUVs (**Figure** **5**). In standard buffer, the presence of NLS in MCMs did not appear to significantly increase their nuclear import compared to non‐NLS‐MCMs (*p* = 0.92, Figure 5a). However, the presence of import buffer significantly increased the nuclear localization of NLS‐DNA‐MCMs (Figure 5a). As expected, import‐promoting factors had a positive effect on NLS‐facilitated translocation, whereas with MCMs lacking NLS, no increase in MCM numbers was observed in the nucleus. Furthermore, the ATTO550‐DNA‐related fluorescence localized inside the nucleus was compared to the number of MCMs in the nucleus, confirming the localization and thus, the successful import of the micelles into the nucleus, where they are likely to release their cargo DNA (Figure 5b,c and S9, Supporting Information).

The Pearson correlation coefficient (PCC) was also used to more accurately describe the correlation between the fluorescence data and the number of MCMs localized in the nucleus. Specifically, the positive PCC of 0.78 (*r* ^2^ = 0.61, Figure 5d) between the DNA‐associated ATTO550 fluorescence and the number of MCMs in the nucleus indicates a strong correlation, suggesting that increased nuclear import of MCMs is accompanied by enhanced ATTO550 fluorescence (Figure 5b,c). Furthermore, given the weak PCC value between the MCMs in the nucleus and its GFP fluorescence intensity (*r* ^2^ of 0.04 and 0.16), no strong correlation could be detected, confirming that the presence of the MCMs did not have an adverse effect on the morphology of the nuclei (Figure S10, Supporting Information). In addition, neither MCMs nor the import buffer had a significantly influenced the volume, sphericity, or GFP fluorescence of the encapsulated nuclei (Figure S11, Supporting Information), as no statistically significant differences were observed across buffer conditions or with NLS presence in the MCMs. These results suggest that nuclear morphology and fluorescence remain stable regardless of buffer composition or MCM presence, indicating that the observed effects are not due to changes in nuclear integrity or GFP expression levels.

## 3 Conclusion

This study presents an advanced bottom‐up strategy to create chimeric protocells that incorporate native cell nuclei within polymeric GUVs. Using double‐emulsion microfluidics, we efficiently produced nucleated protocells, where the morphology and volume of the nucleus is preserved by confinement in a specifically composed cytosol, extending beyond the lifetime of isolated nuclei kept in the corresponding solution. In addition, the nucleus maintained its ability for the NLS‐mediated import of a nucleus‐targeting, multimicellar delivery platform through the nuclear pore complexes embedded in an apparently intact nuclear envelope. Our research demonstrates that by engineering the environment within protocells, we maintained the integrity and the intrinsic GFP expression of the nuclei for at least 20 h at physiological temperature. Significantly, the study demonstrates that protocells containing nuclei inside exhibit a functional import mechanism involving nuclear pore complexes by means of a nanocarrier based on multimicellar peptide displaying nuclear localization sequences. The critical role of the nuclear localization sequence in targeting the nucleus was underscored by the clearly enhanced nuclear translocation of NLS‐exposing MCMs versus MCMs lacking NLS, providing clear evidence of selective nuclear import. Moreover, by providing energy donors and specific transport proteins in the cytosolic environment of the confined nuclei, we significantly improved the efficiency of molecular transport across the nuclear membrane in the encapsulated nuclei. This finding highlights the crucial role of a tailored cytosol in supporting and enhancing cellular functions in bottom‐up constructed protocells. The flexibility in modifying the protocell membrane and cytoplasm opens new avenues not only for the long‐term study of isolated organelles ex vivo but also for the development of semi‐synthetic protocells.

Our proof‐of‐concept study moves nucleated protocells into the realm of reality for unraveling the detailed mechanism behind the NPC transport. It also sets the stage for future research exploring more complex cellular interactions and functionalities in biohybrid systems. In particular, the ease of producing large numbers of protocells harboring native organelles in a confined environment of specific composition by microfluidics opens new avenues for high‐throughput applications in synthetic biology and biotechnology.

## 4 Experimental Section

4.1

4.1.1

##### Reagents

Polyethylene glycol (PEG, *M*_*n*_ = 35 000), polyvinyl alcohol (PVA, MW 13 000‐23 000, 87–89% hydrolyzed), chloroform (99%), anhydrous hexane (95%), sucrose, sodium chloride, Tris‐HCl, CaCl_2_, and MgCl_2_ were obtained from Sigma‐Aldrich. Fmoc‐Trp(Boc)‐OH and Rink Amide AM resin (0.71 mmol g^−1^) were purchased from IRIS Biotech. Ethyl cyano(hydroxyimino)acetate (Oxyma Pure) and all other Fmoc‐protected amino acids were purchased from Novabiochem. Acetonitrile (ACN) and dichloromethane (DCM) were purchased from VWR chemical. Dialysis tubes for solvent exchange were purchased from Spectrum Laboratories (cellulose ester, MWCO 500–1000 Da, 3.2 cm mL^−1^). ATTO550‐labeled 18nt G3139‐GAP DNA was purchased from Microsynth. Trypsin‐EDTA and fetal bovine serum (FBS) were purchased from BioConcept. pluriStrainer Mini 20 μm cell strainers were obtained from pluriSelect. Pluronic F‐68 Non‐ionic Surfactant (100×) and Dulbecco's modified Eagle's medium (DMEM) were purchased from Gibco. BODIPY 630/650 was obtained from Thermo Scientific Inc. Aquapel was obtained from PGW Auto Glass. All chemicals were used as received unless otherwise noted.

##### Cell Culture

Native nuclei were isolated from exponentially growing HeLa cervical cancer cells (Hela GFP‐H2B: Millipore Cat. # SCC117). HeLa and histone H2B‐GFP expressing HeLa were cultured in high glucose Dulbecco's Modified Eagle Medium (DMEM) supplemented with fetal bovine serum (FBS) (10%). All cells were cultured at 37 °C in a humidified incubator with CO_2_ (5%).

##### Nuclei Isolation

Nuclei isolation was carried out using the Minute Detergent‐Free Nuclei Isolation Kit (invent biotechnologies, USA) according to the manufacturer's protocol. In brief, cells were harvested after enzymatic detachment, washed once with ice‐cold PBS and filtered using a 70 μm cell strainer to remove aggregates. Cell pellets were resuspended in buffer A (500 μL) and incubated on ice for 8 min. Sensitized cells were vortexed for 30 s, homogenized with 5 strokes with a handheld Dounce homogenizer and a tight‐fitting pestle, and transferred to a filter cartridge with a collection tube. The extracts were filtered twice by low‐speed centrifugation to rupture the plasma membranes while leaving the nuclei intact. The pellet was resuspended by vortexing for 10 s followed by centrifugation for 3 min at 500 g. The supernatant was removed and the pellet containing nuclei was resuspended in cold buffer B (0.5 mL), centrifuged at 600 g for 9 min to remove residual cell debris. The nuclear pellet was resuspended in assay buffer and filtered using 20 μm cell strainers by centrifugation for 2 s at 600 g. The filtered nuclei were placed on ice and immediately processed.

##### Synthesis of PDMS_*25*_ *‐b‐PMOXA*_*10*_

Synthesis and analysis of the amphiphilic diblock copolymer poly(dimethylsiloxane)_25_‐*block*‐poly(2‐methyl‐2‐oxazoline)_10_ (PDMS_25_‐*b*‐PMOXA_10_) was described elsewhere.^[^ ^44^ ^]^

##### Microfluidic Device Fabrication and Coating

The microfluidic six‐way junction chips were used as published previously.^[^ ^45^ ^]^ Silicon–glass microfluidic chips were etched into silicon wafers at the Binnig and Rohrer Nanotechnology Center at IBM Research Europe–Zurich using deep‐reactive ion etching. A borofloat 33 (BF33) glass cover was used to cover the etched microfluidic by anodically bonding. Chip coating and regeneration was carried out according to a published protocol.^[^ ^20^ ^]^

##### Microfluidic Setup

All fluids were filtered using appropriate hydrophilic or hydrophobic syringe filters (pore size 0.2 μm) prior to passing them through the microfluidic chips. Hamilton gas‐tight syringes (0.5, 1, and 5 mL) with PTFE‐Luer‐lock mechanisms and a Cetoni neMESYS low pressure syringe pump system with 3 modules was used to inject the fluids into the microfluidic chips. Fluorinated ethylene propylene (FEP) tubing (BGB Analytik) with an inner diameter of 0.25 mm (1/16”) were used to connect the syringes to the microfluidic chips.

##### Fluid Phase Composition for Microfluidics

For nuclei encapsulation in microfluidic GUVs, a buffer composed of sucrose (270 mm), Tris‐HCl (10 mm), and MgCl_2_ (1 mm) (281.3 ± 2.1 mOsmol kg^−1^) supplemented with bovine serum albumin (BSA) (0.5%) was used as IA.^[^ ^46^ ^]^ Where indicated, the IA was supplemented with an import promoting buffer at final concentrations of GTP (2 mm), ATP (1 μm), creatine phosphate (4 mm), creatine kinase (20 μg mL^−1^), RanGDP (5 μm), NTF2 (4 μm), and DTT (1 mm). For the OA phase, a solution of PEG (5%, average *M*_*n*_ 35 000), Pluronic F‐68 (0.1%) in PBS (327.7 ± 0.6 mOsmol kg^−1^) was used. A freezing point osmometer (Gonotec Osmomat) was used to measure the osmolarity of the IA and OA phases. The PO phase was composed of PDMS_25_‐*b*‐PMOXA_10_ dissolved (4 mg mL^−1^) in a mixture of hexane and chloroform (3:2 v/v).

##### Double Emulsion Fabrication and Dewetting

PO, IA, and OA phases were flowed at flow rates of 1, 3, and 5 μL min^−1^ respectively into the microfluidic chip. The formed double emulsions were collected for 10 min in an 1.5 mL Eppendorf tube containing OA (300 μL). Evaporation of the organic phase yielded ≈10^6^ GUVs at a concentration of ≈10^6^ mL^−1^.^[^ ^20^, ^45^ ^]^

##### Nuclei Encapsulation

Nuclei were encapsulated by adding freshly isolated nuclei to the IA at a 1:5 dilution ratio, resulting in a concentration of ≈10^6^ nuclei mL^−1^.

##### Multicompartment Micelle Synthesis and Assembly

Detailed procedures for MCM synthesis and assembly are described in refs. [30, 31]. In brief, the amphiphilic NLS‐peptide (KR)_2_(HR)_2_gT consisting of 23 amino acids (H_2_N–[K–R]_2_‐[H–R]_2_–[W–_D_L]_7_–W–NH_2_, with _D_L = D‐Leucine) and the nonNLS‐peptide (HR)_3_gT consisting of 19 amino acids (H_2_N–[HR]_3_[W–_D_L]_6_–W–NH_2_) were synthesized using an automated microwave peptide synthesized (Liberty Blue, CEM, Germany). The synthesized peptides were purified by reversed phase high performance liquid chromatography (RP‐HPLC) (Prominence 20 A, Shimadzu, Japan) on a C18‐TSE (VDSpher OptiBio PUR 300 C18‐TSE, 20 × 250 mm, VDS Optilab, Germany) column and the molecular mass was determined by PerSeptive Biosystems Voyager‐DE‐PRO time‐of‐flight mass spectrometer (MALDI‐ToF‐MS) in positive mode.^[^ ^30^ ^]^ Peptide fractions were lyophilized and stored at −20 °C.

The amphiphilic peptides were self‐assembled by dissolving them (1 mg mL^−1^) in a mixture of ethanol and water (1:1, v/v). Self‐assembly was done by solvent exchange, that is, dialyzing the ethanol against MilliQ H_2_O. DNA‐loading of the MCMs was done by adding the 5' ATTO550 labeled 18 nucleotide G3139‐GAP antisense oligonucleotide (ASO) sequence (SI) (3 μg) to DNA stock solution (100 μL at 1 mg mL^−1^). The DNA‐peptide mixtures were then diluted to a final volume (500 μL) and final ethanol concentration (35%) and dialyzed at 4 °C for 20 h in a prewashed 500–1000 MWCO dialysis tube. Dialysis took place against ultrapure DNase/RNase‐free distilled water (500 mL).

##### Multicompartment Micelle Characterization

All MCMs, self‐assembled from NLS‐peptide or nonNLS‐peptide, with or without DNA, were analyzed from a colloidal perspective.

The mean hydrodynamic diameter and polydispersity index (PDI) of MCMs were determined at 25 °C by DLS using a Zeta Sizer Nano ZSP (Malvern Instruments Ltd, UK) instrument at a wavelength of 633 nm with an angle detection of *θ* = 173°. Samples were diluted (1:20) in MilliQ water for DLS measurements. All measurements were performed in triplicate. For measuring the zeta‐potential of MCMs, a cuvette was filled with MCMs diluted in water, and the zeta potential recorded after each polyelectrolyte deposition. Zeta potential data represent the mean of three consecutive measurements. The nanoparticle tracking analysis (NTA) of MCMs was performed using a NanoSight NS300 instrument (Malvern Panalytical). First, MCMs were diluted 20‐fold and applied to the viewing chamber using a 1 mL syringe. Nanoparticle movement was analyzed by the NTA software (version 3.4, NanoSight) based on tracking each particle on a frame‐by‐frame basis. For each measurement, three videos of 60 s were captured at room temperature and the software provided the mean and median particle size together with the estimated concentration of the MCMs in solution. The entrapment of ATTO550‐labeled 18 nucleotide G3139‐GAP antisense oligonucleotide in MCMs was previously described. (Tarvirdipour).

##### Cryogenic Electron Microscopy

Four microliters of MCMs were adsorbed onto glow discharged carbon‐coated grids (Lacey, Tedpella, USA). Excess sample was blotted off with grade 1 Whatman filter paper for seconds to produce a thin aqueous film which was subsequently vitrified by plunge freezing. Frozen grids were transferred at −178 °C into a Gatan 626 cryoholder and imaged by Talos electron microscope (FEI, USA). Electron micrographs were recorded at an accelerating voltage of 200 kV while keeping the sample at low temperatures. Resulting cryo‐EM micrographs were recorded on a CETA camera (4096 × 4096 pixels; Thermo Fisher).

##### Nuclei Targeting Using MCMs in GUVs

MCM nanocarriers were diluted (1:10) to a final volume of ≈4.29 × 10^7^ ± 5.4 × 10^6^ particles mL^−1^ and combined with the nuclei in the IA. All samples were kept on ice until applied to the microfluidic setup.

After GUV formation, nuclei‐GUVs were incubated for 4 h at room temperature before imaging, unless otherwise stated.

##### Nuclei‐GUV Imaging

For analysis by confocal laser scanning microscopy (CLSM), nuclei‐encapsulating GUVs were typically diluted (1:4) in OA and applied to an Ibidi μ‐slide. The GUV membranes were stained with BODIPY 630/650 hydrophobic membrane selective dye unless indicated otherwise (2.5 μm). A Zeiss 880 confocal laser scanning microscope (Zeiss, Germany) equipped with a water‐immersion C‐Apochromat 40×/1.2 objective was used for image acquisition. A 488 nm argon laser, a 561 nm DPSS 5561‐10 laser, and a 633 nm HeNe laser were used to excite fluorophores. Images were typically recorded in super‐resolution Airyscan mode and processed using the Zeiss ZEN software (Version 2.3). The images were analyzed using Fiji image analysis software and Python scripts to evaluate GUV fluorescence or size.

##### 3D Reconstruction of Nuclei‐Encapsulating GUVs

3D image reconstruction from Airyscan z‐stack images was performed using Imaris software (Bitplane). In summary, the z‐sections were 3D reconstructed in Imaris followed by gamma correction, background reduction and nucleus segmentation based on the GFP fluorescence intensity, resulting in a surface representing the nucleus. Nucleus segmentation was performed as a batch process but was individually adjusted for each nucleus to improve surface detection.

The morphological and fluorescent data was then exported as csv files for subsequent processing. In a second step, the MCMs were detected using the “Spots” workflow with identical sensitivity parameters for all samples within an experiment, resulting in MCM localization in 3D space and relative to the nucleus surface. The MCM localization data was also exported as csv files. The image analysis workflow is described in Figure SX. The csv files were then analyzed using Python scripts. For the determination of MCM inside the nucleus, the nuclear envelope was defined as −0.2 to +0.1 μm from the segmented nuclear surfaces.

##### Data Processing

The data was processed using Python. No data pre‐processing was done prior to analysis. For MCM localization plotting (e.g., Figure S3, Supporting Information), the distance data was smoothed using a moving average with a window size of 15 datapoints.

##### Fluorophore Correlation

The Pearson correlation coefficient (PCC) of two fluorophore data sets *X* and *Y* was calculated per nucleus with the following formula:

(1) $${\text{PCC}}_{X,Y}=\frac{\text{cov}\left(X,Y\right)}{\sigma_{X}\sigma_{Y}}$$

where cov is the covariance, *σ*_*X*_ is the standard deviation of *X* and *σ*_*Y*_ is the standard deviation of *Y*.

##### Statistical Analysis

Two‐way Analysis of Variance (ANOVA) was used for comparative analysis, followed by the post‐hoc Tukey's honestly significant difference (HSD) test. Before statistical analysis, data were tested for normal distribution using the Shapiro–Wilk test. *p* < 0.05 was considered statistically significant. The significance level of the calculated *p* values was indicated using asterisks: *p* > 0.05 (n.s.), *p* < 0.05 (*), *p* < 0.005 (**), and *p* < 0.0005 (***). In boxplots, the center line depicts the median and boxes span the interquartile range (25–75%). Outliers are indicated in the dot plots or using rhombuses.

## Conflict of Interest

The authors declare no conflict of interest.

## Author Contributions

**Lukas Heuberger**: conceptualization (equal); methodology (equal); visualization (lead); writing—original draft (lead); writing—review & editing (equal). **Arianna Balestri**: investigation (equal); methodology (equal); visualization (equal); writing—review & editing (equal). **Shabnam Tarvirdipour**: investigation (equal); methodology (equal); visualization (supporting); writing—review & editing (supporting). **Larisa E. Kapinos**: methodology (supporting); writing—review & editing (supporting). **Roderick Y. H. Lim**: conceptualization (supporting); writing—review & editing (supporting). **Emanuel Lörtscher**: resources (lead); writing—review & editing (supporting). **Cora‐Ann Schoenenberger**: conceptualization (lead); methodology (equal); visualization (equal); writing—review & editing (lead). **Cornelia G. Palivan**: conceptualization (equal); funding acquisition (lead); writing—review & editing (equal). **Lukas Heuberger** and **Arianna Balestri** contributed equally to this work.

## Supporting information

Supplementary Material

## Data Availability

The data that support the findings of this study are openly available in [zenodo] at [https://doi.org/10.5281/zenodo.14168164], reference number [14168164].
