# Supplementary material for: Polymeric Giant Unilamellar Vesicles Support Longevity of Native Nuclei in Protocells
Source: Small Sci. 2025 Mar 18;5(6):2400622. doi: 10.1002/smsc.202400622 (PMC12168613; doi:10.1002/smsc.202400622)
Supplement: Supplementary file 1 — Supplementary Material [file SMSC-5-2400622-s001.pdf]

Supporting Information

Polymeric GUVs Support Longevity of Native Nuclei in Protocells

*Lukas Heuberger[+], Arianna Balestri[+], Shabnam Tarvirdipour, Larisa Kapinos, Roderick Y. H. Lim, Cora-Ann Schoenenberger, Cornelia Palivan**

1. Supplementary Methods

Estimation of Encapsulation Efficiency

The number of encapsulated nuclei per GUV (N_{nuc}) was calculated based on the volume of the GUV (V_{GUV} in L) and the nuclei concentration (c_{nuclei}):

$$N_{nuc} = c_{nuclei} \times V_{GUV}$$

The GUV volume was calculated as $V_{GUV} = \frac{4}{3} r_{GUV}^3 \pi$.

2. Supplementary Tables

Table S1. Comparison of morphology and fluorescence characteristics of nuclei *in cellulo*, isolated and encapsulated in pGUVs.

Nuclei	Incubation Time [h]	Volume [µm^3]	Sphericity [a.u.]	GFP Fluorescence [a.u.]
**in cellulo**	-	803 ± 217	0.85 ± 0.05	4604 ± 1743
**isolated**	4	1796 ± 693	0.87 ± 0.07	1493 ± 583
**isolated**	24	890 ± 285	0.51 ± 0.16	162 ± 161
**in pGUV**	4	675 ± 437	0.84 ± 0.15	6913 ± 2403
**in pGUV**	24	465 ± 203	0.79 ± 0.18	6332 ± 3472

Table S2. GUV diameter and osmolarity difference between inner aqueous (IA) and outer aqueous (OA) phase.

Buffer	GUV diameter [µm]	$\Delta_{Osmolality}$ (OA-IA) [mOsmol kg^{-1}]
**standard buffer**	38.5 ± 0.9	46.4
**import buffer**	37.4 ± 0.8	15.7

3. Supplementary Figures

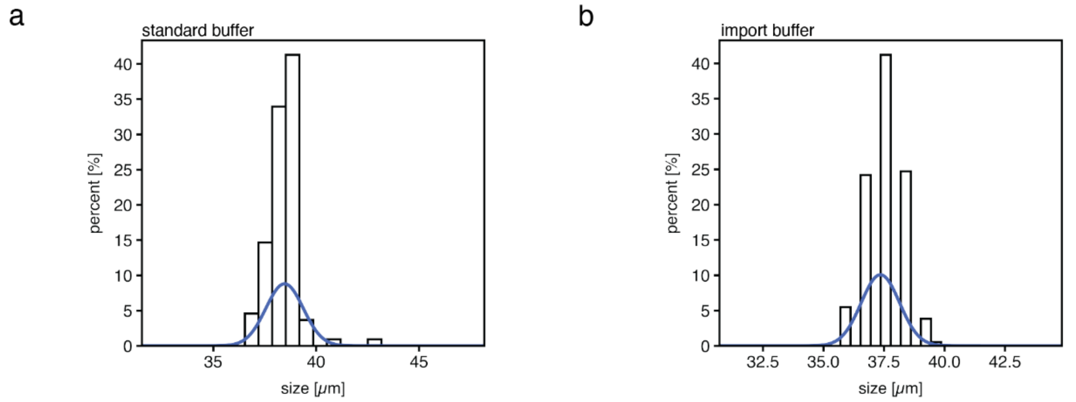

Figure S1 – Influence of buffer on GUV size. Size histograms and regressed normal distribution (blue line) of GUVs made with (a) standard buffer and (b) import buffer. Average diameters are (a) 38.5 ± 0.9 μm in standard buffer and (b) 37.4 ± 0.8 μm in import buffer (n > 100), also presented in Table S2.

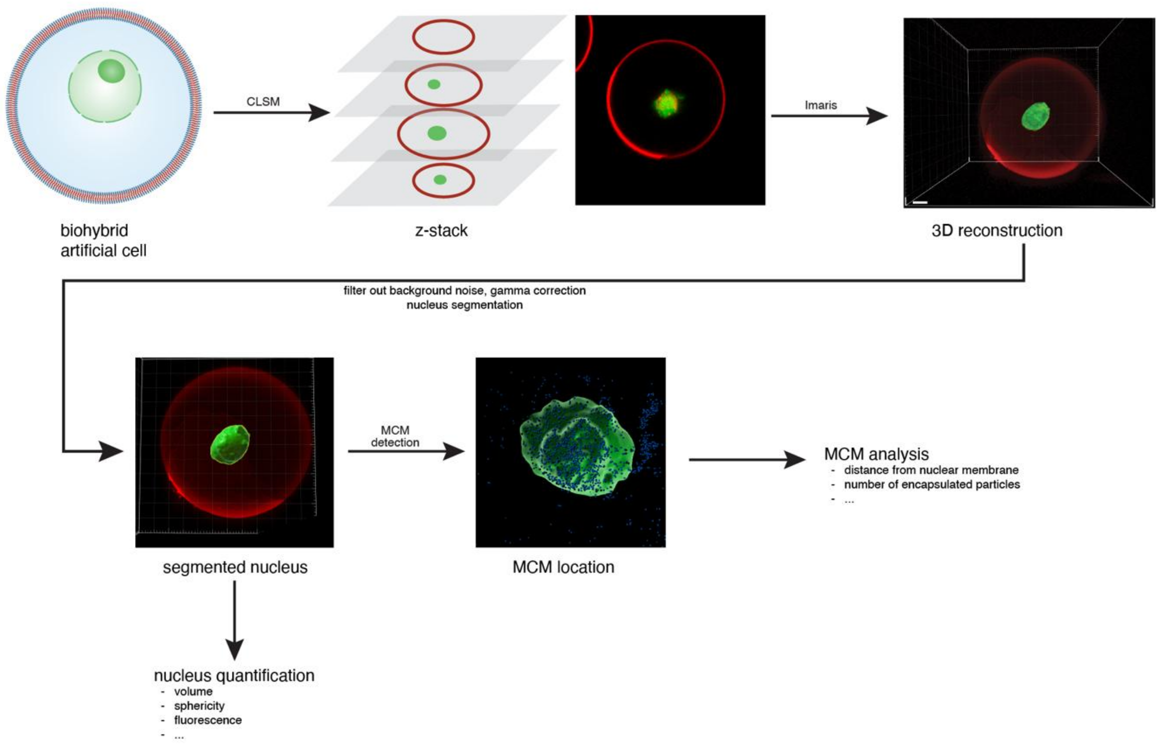

Figure S2 - Nuclei-pGUV analysis workflow. Nuclei-encapsulating GUVs are imaged by CLSM as image stacks along the z axis of the whole GUV or of the encapsulated nucleus. A 3D-reconstruction is generated using Imaris microscopy image analysis software. After filtering and background correction of the fluorescent data, the nucleus is segmented based on its GFP fluorescence. The volume, fluorescence, and sphericity are calculated from the

nucleus segmentation. In a next step, the MCMs are detected in the image based on their ATTO550-fluorescence. Based on the GFP fluorescence of the nucleus and the MCM location, the distance of the MCMs from the nuclear surface is calculated, and the distribution of MCMs is determined relative to the total number inside the MCM-nucGUVs.

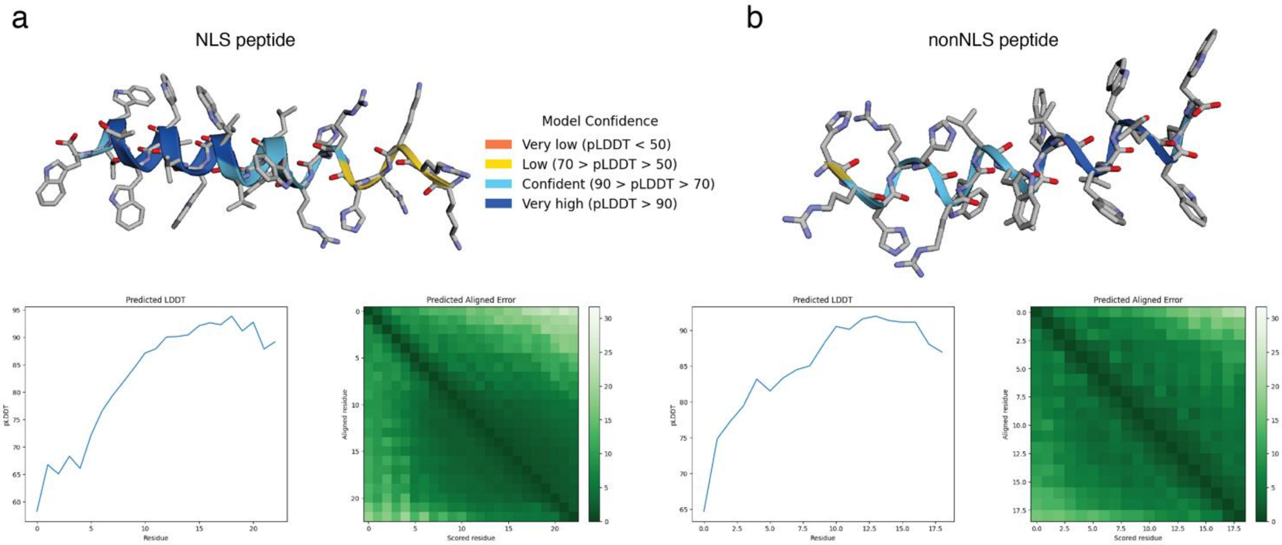

Figure S3 - 3D structure of NLS- and nonNLS-peptides. 3D structure and per-residue model confidence score (pLDDT) of (a) NLS-, and (b) nonNLSpeptides. Structure prediction made with AlphaFold1.

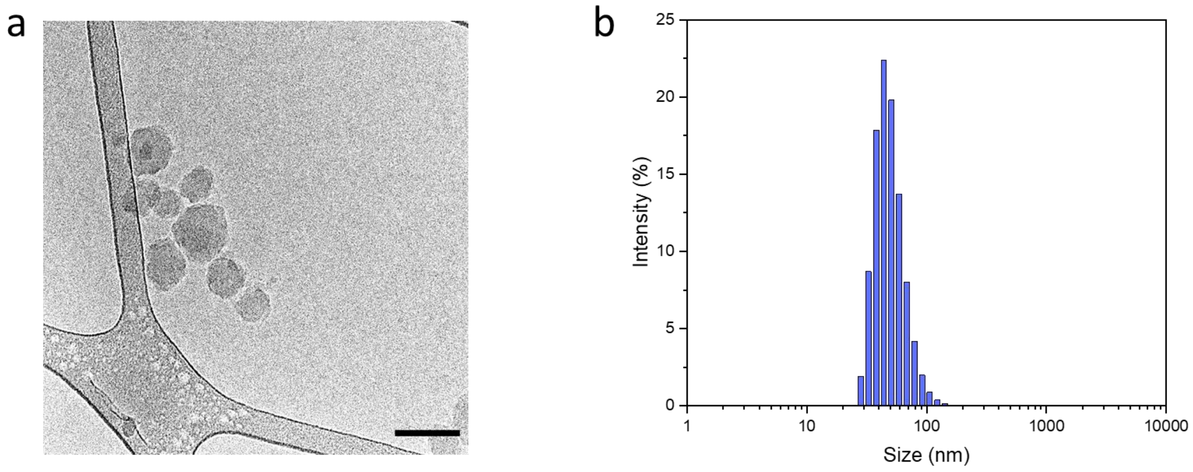

Figure S4 – Physicochemical characterization of self-assembled NLS-DNA-MCM nanocarriers. (a) Cryo-TEM, and (b) Size distribution by DLS. Scale bar 100nm.

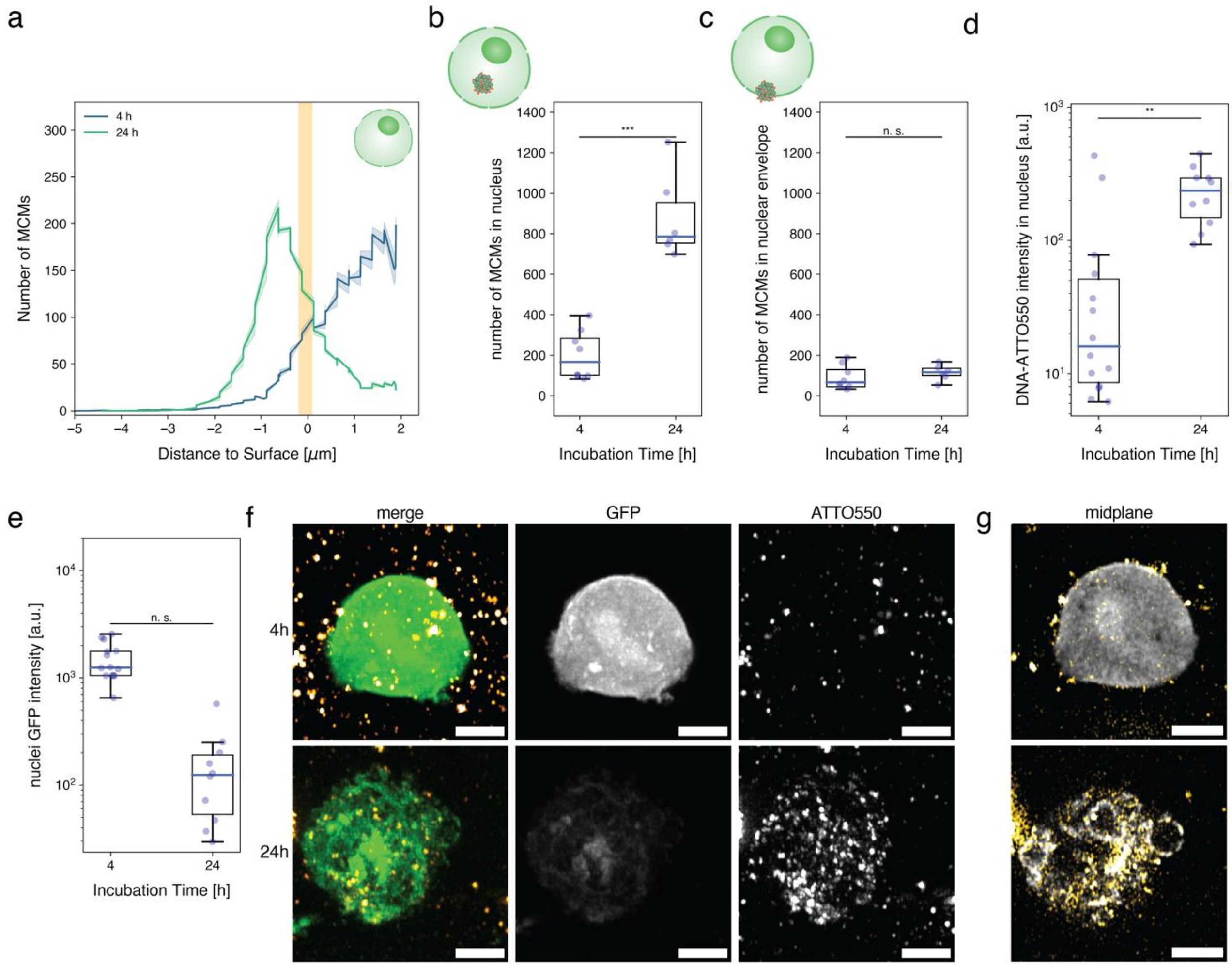

Figure S5 - Targeting MCMs to isolated nuclei. a, MCM localization respective to the nuclear surface after 4 h or 24 h incubation. The nuclear envelope region is indicated in orange. b, Number of MCMs within isolated nuclei after 4 h or 24 h of incubation. c, Number of MCMs close to the nuclear envelope after 4 h or 24 h of incubation. d, DNA-associated ATTO550 fluorescence of nuclei after 4 h or 24 h incubation. e, GFP fluorescence of nuclei after 4 h or 24 h incubation. n≥10 nuclei/condition. One-way ANOVA was used for comparison: $p > 0.05$ (n.s.), $p < 0.05$ (*), $p < 0.005$ (**), $p < 0.0005$ (***), and n.s. = not significant, Tukey's post hoc test. f, Representative micrographs depicting maximal projections along the z axis of

GFP-fluorescent nuclei (green) and ATTO550-loaded MCMs (yellow) over time. Single channels are depicted in black and white. g, Mid-plane images of nuclei depicted in (f) showing the localization of the MCMs (yellow) with respect to the cross-sectional area of the nucleus (black/white). Scale bars, 5 µm.

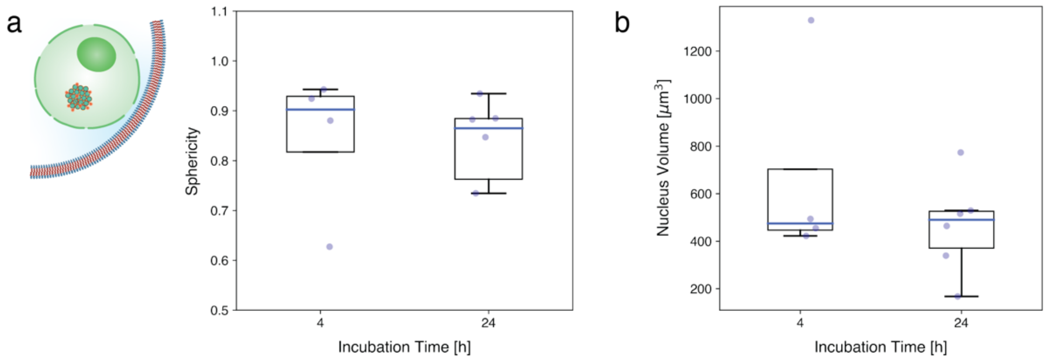

Figure S6 - Sphericity and volume of encapsulated nuclei after different incubation times. Mean sphericity (a) and volumes (b) of nuclei after 4 and 24 h incubation with MCMs.

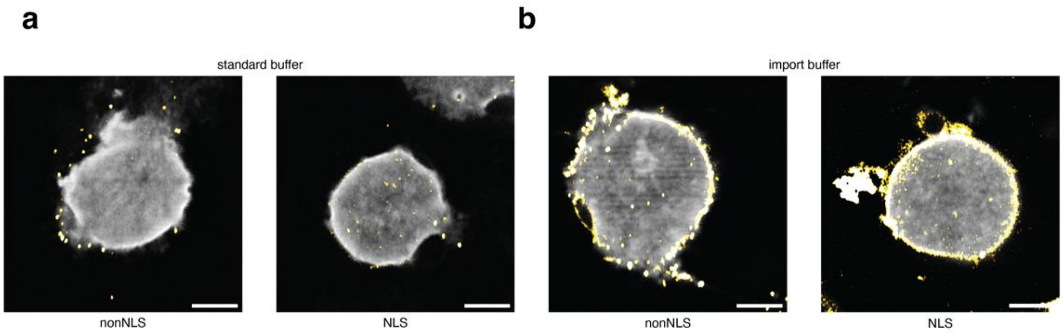

Figure S7 – Mid-plane optical sections of encapsulated nuclei. Cross sections at maximum width of nuclei represented in 3D in Figure 4e (a) and 4f (b). GFP-fluorescence of nuclei is shown in white and ATTO550 fluorescence of ATTO550-loaded MCMs in yellow. Scale bars, 5 µm.

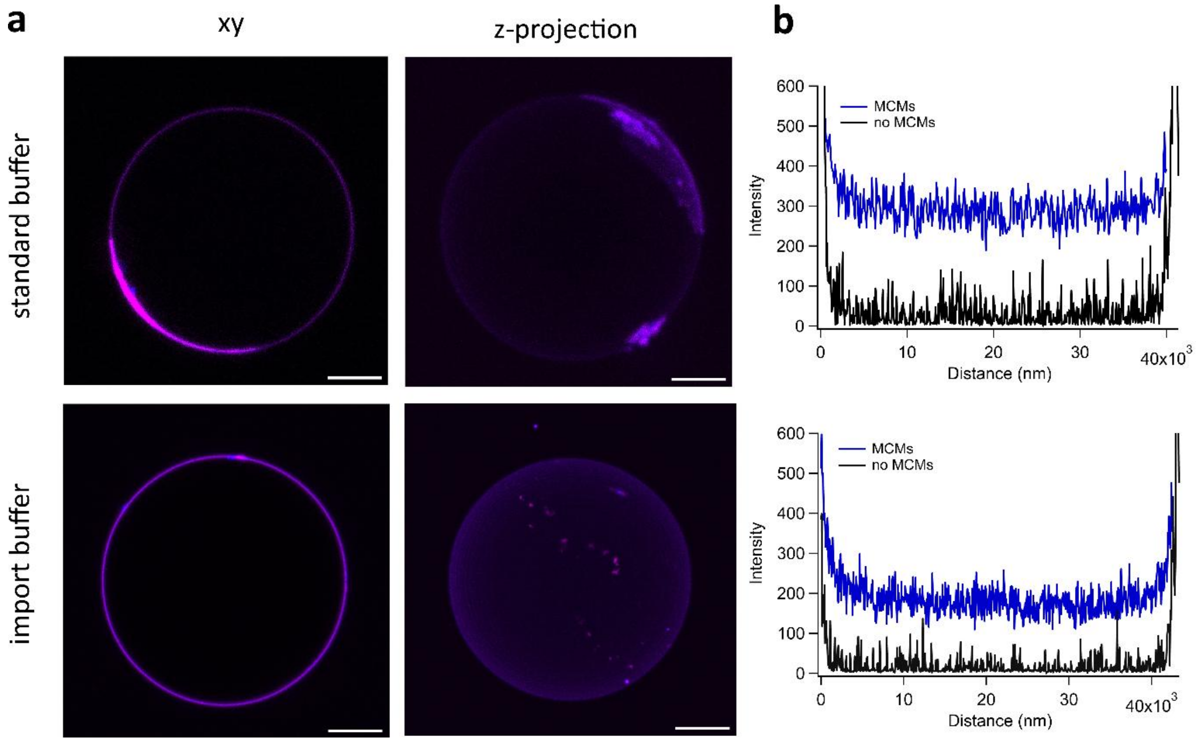

Figure S8 – Stability of MCMs inside pGUVs. a, Representative micrographs of ATTO550-DNA-loaded MCMs (blue) and BODIPY 630/650 stained GUVs (red), displayed in xy-plane (xy) and along the z axis (z-projection) in standard (upper part) and import buffer (lower part) 24h post-encapsulation by microfluidics. b, fluorescence intensity profile of ATTO550-DNA-loaded MCMs inside GUVs in standard (upper part) and import buffer (lower part) 24h post-encapsulation by microfluidics.

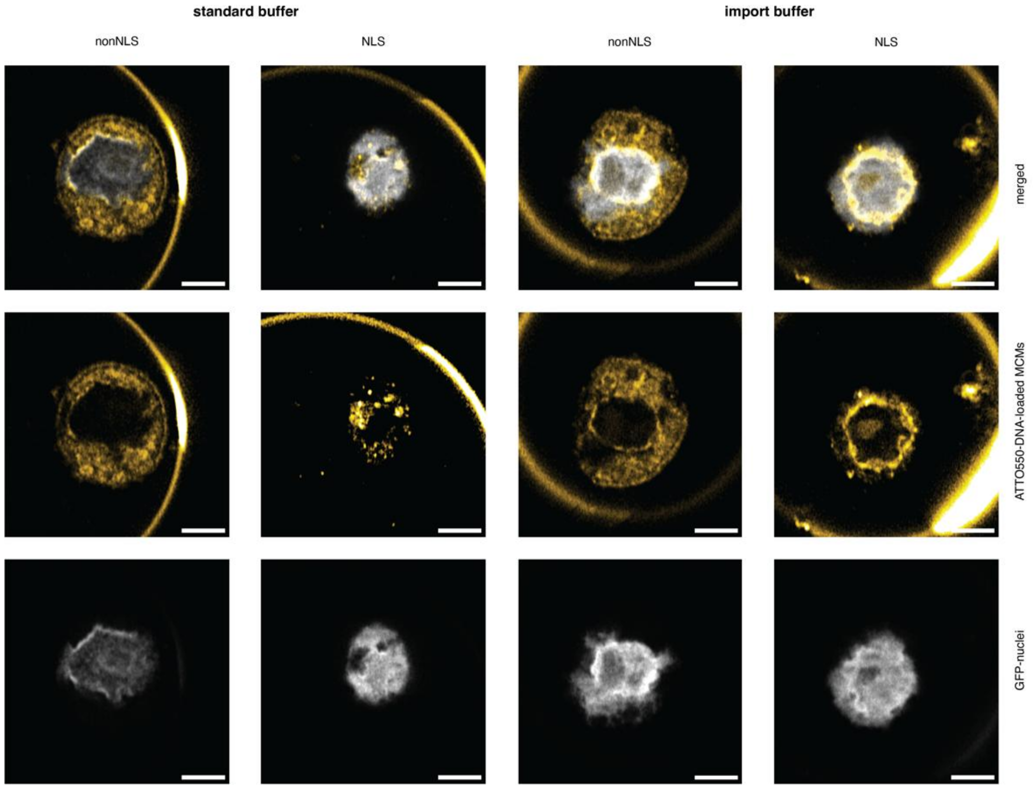

Figure S9 – Nucleus cross sections. Mid-plane images of encapsulated nuclei shown as 3D renderings in Figure 5c. MCM-nucGUVs encapsulating ATTO550-DNA-loaded MCMs. GFP-fluorescence of nuclei is shown in white and ATTO550 fluorescence of ATTO550-loaded MCMs in yellow. Scale bars, 5 μm.

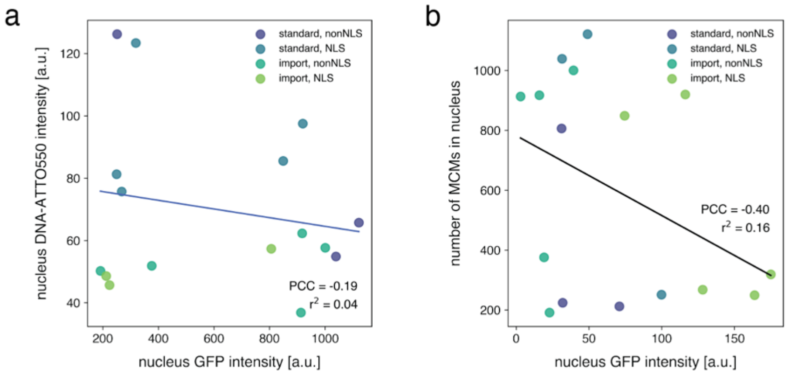

Figure S10 - Correlation between nucleus fluorescence and number of MCMs in the nucleus. a, Correlation between GFP fluorescence and ATTO550 fluorescence of the nucleus. Linear regression (blue line), r^2 = 0.04, Pearson correlation coefficient = -0.19. b, Correlation

between GFP fluorescence of the nucleus and number of MCMs in the nucleus. Linear regression (blue line), r^2 = 0.16, Pearson correlation coefficient = -0.4.

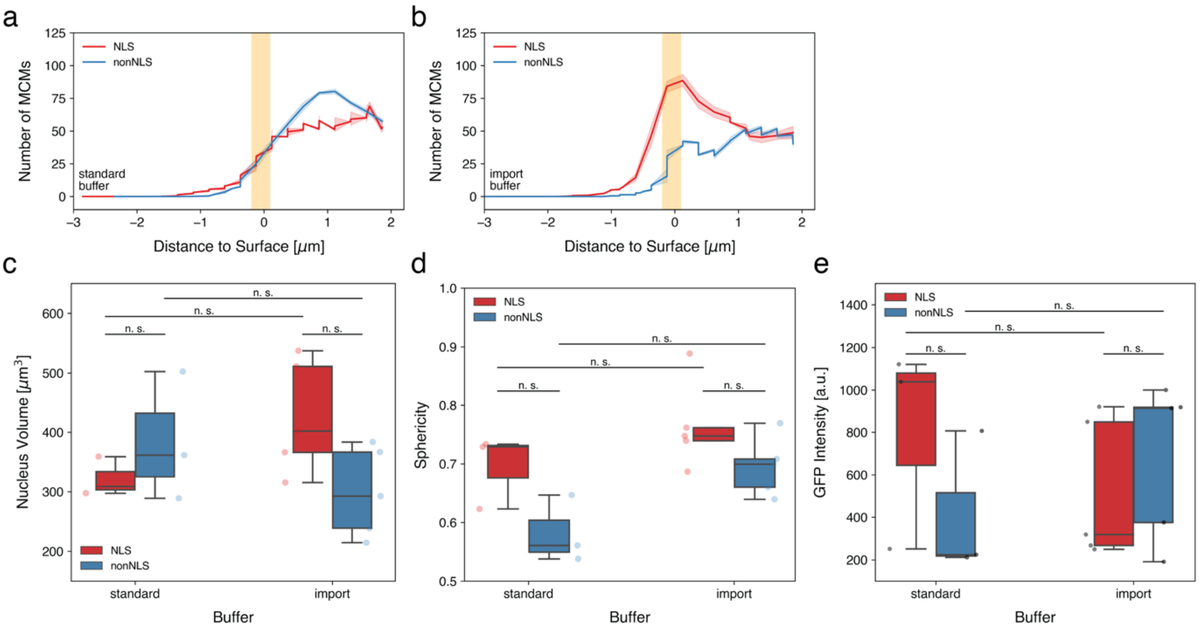

Figure S11 - MCM localization and nucleus morphology in MCM-nucGUVs. a,b, MCM localization respective to the nuclear surface of nuclei encapsulated in pGUVS in (a) standard and (b) import buffer. The nuclear envelope region is indicated in orange. c, Volume of encapsulated nuclei co-encapsulated in standard and import buffer with either nonNLS- or NLS-MCMs. d, Sphericity of encapsulated nuclei co-encapsulated with either nonNLS- or NLS- MCMs in standard and import buffer. e, GFP fluorescence intensity of encapsulated nuclei in standard and import buffer co-encapsulated with nonNLS- and NLS-MCMs.

3. Supplementary Notes

1. Peptide Sequences

Amino acid sequences for nanoparticles used for nuclei targeting:

**NLS peptide**	$KR_2(HR)_2gT$	KRKRHRHRWLWLWLWLWLWLWLW
**nonNLS peptide**	$(HR)_3gT$	HRHRHRWLWLWLWLWLWLW

2. DNA Sequences

DNA sequences for nanoparticles used for nuclei targeting:

ATTO550-G3139-GAP ATTO550-5'-ucu CCC AGC GTG CGC cau-3'

Lower case indicates 2'-OMe modification.

3. MCM targeting in isolated nuclei

In isolated nuclei, the number of MCMs associated with the nucleus clearly increased over time. The localization of the MCMs was distinguished between MCMs within the nucleus and those associated with the nuclear envelope, defined as the region extending from 0.2 µm inside to 0.1 µm outside the detected nuclear membrane (orange region). The total number of MCMs within the nuclei increased significantly with the incubation time (Figure S5), while the particles in the membrane region of the nuclei remained similar with time (Figure S5), indicating that the NLS-displaying MCMs are continuously taken up by the isolated nuclei. Although the nuclear localization of MCMs was assessed by fluorescence of entrapped ssDNA, MCMs have been shown to disintegrate over time,[1,2] which could bias their detection in both directions; aggregated MCMs may be too large to pass through the NPCs, whereas disintegration of individual micelles may result in smaller MCMs that are more

readily imported into the nucleus. This requires not only the quantification of the MCMs but also the ATTO550 fluorescence of the whole nucleus, which has also been shown to increase significantly (Figure S5). Although the number of MCMs inside the nucleus increased over 24 hours, the nuclear GFP fluorescence of HeLa-H2B-GFP cells decreased significantly (Figure S5). This data suggested that due to the deterioration of the nucleus and possible disassembly of the nuclear envelope, MCMs more readily translocate into the nucleus. Thus, to maintain a good balance between viability and adequate incubation time, subsequent experiments were analyzed 4 h post encapsulation by microfluidics.

References

[1] S. Tarvirdipour, M. Skowicki, C.-A. Schoenenberger, L. E. Kapinos, R. Y. H. Lim, Y. Benenson, C. G. Palivan, *Biomater. Sci.* **2022**, *10*, 4309.

[2] S. Tarvirdipour, M. Skowicki, V. Maffeis, S. N. Abdollahi, C.-A. Schoenenberger, C. G. Palivan, *Journal of Colloid and Interface Science* **2024**, *664*, 338.
